# Pre-treatment tumour perfusion parameters and initial RECIST response do not predict long-term survival outcomes for patients with head and neck squamous cell carcinoma treated with induction chemotherapy

**DOI:** 10.1371/journal.pone.0194841

**Published:** 2018-03-28

**Authors:** Natalie M. Lowe, Lucy E. Kershaw, Jonathan M. Bernstein, Stephanie B. Withey, Kathleen Mais, Jarrod J. Homer, Nicholas J. Slevin, Suzanne C. Bonington, Bernadette M. Carrington, Catharine M. West

**Affiliations:** 1 Division of Cancer Sciences, Manchester Academic Health Science Centre, University of Manchester, The Christie NHS Foundation Trust, Manchester, United Kingdom; 2 Christie Medical Physics and Engineering, The Christie NHS Foundation Trust, Manchester, United Kingdom; 3 Department of Otolaryngology—Head & Neck Surgery, The Royal Marsden NHS Foundation Trust, London, United Kingdom; 4 Medical Physics, University Hospitals Birmingham, Birmingham, United Kingdom; 5 Head and Neck Clinical Oncology, The Christie NHS Foundation Trust, Manchester, United Kingdom; 6 University Department of Otolaryngology—Head & Neck Surgery, Manchester Academic Health Science Centre, Manchester University NHS Foundation Trust, Manchester, United Kingdom; 7 Department of Radiology, The Christie NHS Foundation Trust, Manchester, United Kingdom; 8 NIHR Manchester Biomedical Research Centre, Manchester Academic Health Science Centre, Central Manchester University Hospitals NHS Foundation Trust, Manchester, United Kingdom; The Ohio State University, UNITED STATES

## Abstract

**Objectives:**

Previously, we showed that pre-treatment tumour plasma perfusion (F_p_) predicts RECIST response to induction chemotherapy (ICT) in locoregionally advanced head and neck squamous cell carcinoma (HNSCC). The aim here was to determine whether the pre-treatment tumour F_p_ estimate, changes in tumour F_p_ or RECIST response post 2 cycles of ICT were prognostic for long-term survival outcomes.

**Methods:**

A prospective study enrolled patients with high stage HNSCC treated with docetaxel (T), cisplatin (P) and 5-fluorouracil (F) (ICT) followed by synchronous cisplatin and intensity modulated radiotherapy. Dynamic contrast-enhanced magnetic resonance imaging (DCE-MRI) before and after two cycles of ICT was used to measure F_p_ and RECIST response.

**Results:**

Forty-two patients were recruited and 37 underwent two scans. The median follow-up was 36 (range 23–49) months. Pre-treatment tumour F_p_ (stratified by median) was not prognostic for overall survival (p = 0.42), disease specific survival (p = 0.20) and locoregional control (p = 0.64). Neither change in tumour F_p_ nor RECIST response post two cycles of ICT was prognostic for any outcome (p>0.21).

**Conclusion:**

DCE-MRI parameters do not predict long-term survival outcomes following ICT and RECIST response to ICT may not be an appropriate endpoint to determine early efficacy of a treatment in HNSCC patients.

## Introduction

Head and neck cancer is one of the world’s leading malignancies with an estimated global incidence of over 686,000 cases in 2012. In Europe in 2013, head and neck cancer contributed 135,400 new oncology diagnoses and 61,300 deaths.[1] Concurrent chemoradiotherapy is the non-surgical standard of care for patients who present with high stage disease.[2–4] Despite advances in chemoradiotherapy, unlike some other cancer sites where survival rates rose substantially in recent decades, the improvement in head and neck cancer survival rates has been modest. As chemotherapy primarily acts as a radiosensitiser for locoregional treatment in head and neck squamous cell carcinoma (HNSCC),[5] the use of induction chemotherapy (ICT) has been explored to tackle distant metastases.[6] The preferred ICT regimen comprises a taxane (T), platinum agent (P) and 5-fluorouracil (F) (TPF) [7–10] and has been shown in several studies to lower distant metastasis rates compared with CRT alone.[5, 7, 11–13] Despite this, there is controversy as to whether this translates to an improved overall survival outcome.[14, 15] It has been suggested, however, that as well as several problems with poor methodology undermining the applicability of trials comparing ICT to CRT alone; trials have included patients that are unlikely to benefit from the potential advantages of ICT such as those with a low risk of distant metastases, hence diluting any positive effects.[14] Tumour heterogeneity also affects response to treatment.[16] The key to extracting the benefits of ICT may be meticulous patient and tumour selection.

Three cycles of TPF take nine weeks to complete. Approximately 30% of patients do not respond to ICT and hence may have their definitive treatment delayed for little if any benefit.[8, 17] Methods to detect prior to or early in the course of ICT which patients are unlikely to respond would identify patients who should be directed immediately to CRT to prevent delays in definitive locoregional treatment.

Zima et al [18, 19] showed that HNSCCs with elevated blood volume and blood flow detected by pre-treatment computed tomography (CT) perfusion imaging had a good response to ICT. Petralia et al [20] also found baseline tumour blood volume in patients with upper aerodigestive tract squamous cell carcinomas was significantly lower in non-responders to ICT as demonstrated by perfusion CT. Our group set up a study to investigate whether similar findings were seen with dynamic contrast-enhanced magnetic resonance imaging (DCE-MRI), and showed that pre-treatment tumour plasma perfusion (F_p_) predicts response to ICT.[17] Whether the prediction of early response to ICT relates to long-term survival outcomes, however, is not known. This study therefore investigated whether the pre-treatment tumour F_p_ estimate, plus changes in tumour F_p_ or RECIST response post 2 cycles of ICT were prognostic for long-term survival outcomes.

## Materials and methods

### Patients

Ethical approval was granted by The North West 1 Research Ethics Committee (ref: 11/H1017/5) and The Christie Research and Development Department for a prospective open cohort study to recruit 50 patients. Recruitment started in March 2011 and the 50^th^ patient was recruited in July 2013. All patients gave written informed consent. Patients were eligible if they had histologically or cytologically proven stage IV HNSCC (staged according to American Joint Committee on Cancer (AJCC) using the tumour, node, metastases (TNM) system [21]) and had been referred for treatment with three cycles of ICT followed by CRT as decided by The Christie Head and Neck multidisciplinary team. ICT was a modified version of the TAX 323 doses [7] planned as three cycles of: docetaxel (75 mg/m^2^ IV on day 1), cisplatin (75 mg/m^2^ IV on day 1) and 5-FU (750 mg/m^2^ IV on days 2–5) followed by two weeks of rest. Following the two-week rest period after the third cycle of TPF, patients were given concurrent CRT involving intensity-modulated radiation therapy (IMRT) with 66 Gy in 30 fractions plus concurrent cisplatin (100 mg/m^2^ IV) at the beginning of week 1 and week 4 (day 1 and 22). It has been suggested that ICT and CRT provide different benefits for advanced HNSCC with CRT mainly producing locoregional control and ICT controlling distant metastases hence complimenting each other’s strengths.[5] As part of this study was to understand which patients may or may not benefit from ICT as an neoadjuvant to the current gold standard CRT treatment and as efforts to determine a standard CRT regimen post ICT are ongoing separately [6]; we used this well recognised chemoradiotherapy regimen suitable for use as a primary therapy without ICT as per Pignon 2009.[5] Patients were excluded from the trial if they had undergone any previous treatment for a head and neck carcinoma. P16 status was determined as described elsewhere.[17]

### Imaging protocol

Baseline imaging was acquired in the three weeks before ICT. The imaging protocol is described in detail elsewhere.[17] Briefly, the examination consisted of conventional staging scans followed by high resolution T_2_-weighted (w) imaging, a saturation-recovery measurement of T_1_, and dynamic T_1_w imaging for a total of 7 minutes during which a bolus injection of 0.1 mmol/kg Gadobutrol (Gd) was administered in an antecubital vein. DCE-MRI analysis was on a whole-tumour region of interest basis (outlined on high resolution T_2_w images by two radiologists in consensus). The two compartment exchange model [22] was fitted to the Gd concentration vs time curves, which were converted from signal intensity vs time curves using a precontrast measurement of T1.[23] Arterial input functions were obtained from the internal carotid artery on a patient by patient basis using an automated procedure.[24]

A further DCE-MRI examination was acquired during the two-week window between the second and third cycles of ICT. F_p_ of the primary tumour and largest regional lymph node was determined on the second scan. Patients were divided into complete responders, partial responders and those with stable disease according to the Response Evaluation Criteria in Solid Tumours (RECIST 1.1).[25]

### Study design

**Fig 1:**
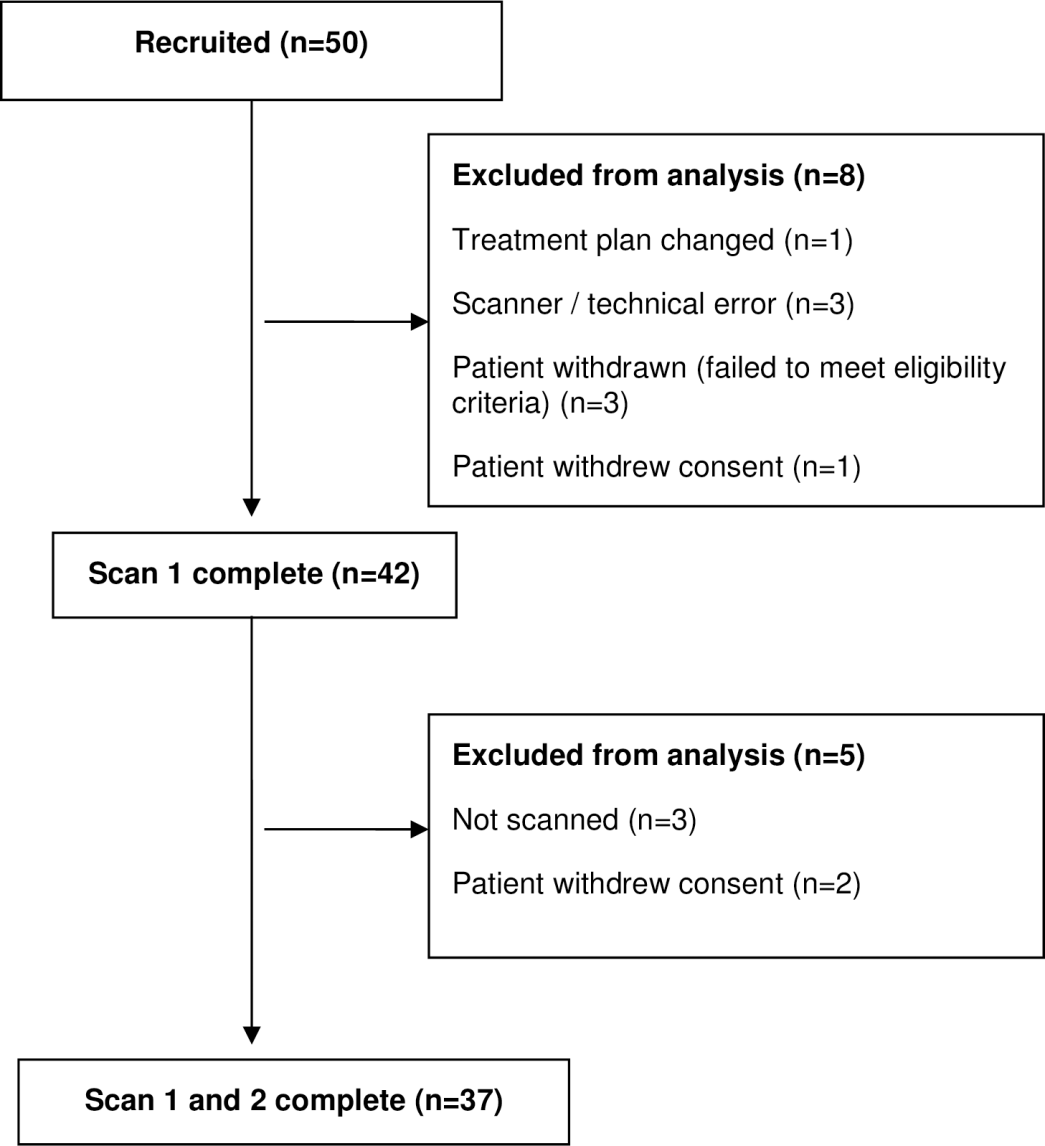
Flow diagram showing patient inclusion and exclusion from the trial.

The prospective study was designed and powered to detect early response to ICT based on published data. The power calculation indicated a need for 38 patients to have a pre-treatment DCE-MRI scan to detect a one standard deviation difference in microvascular parameters with 77% power. Fifty patients were recruited to the trial and 42 completed the baseline DCE-MRI scan. Thirty-seven patients completed both the baseline DCE-MRI scan and the follow-up scan after two cycles of ICT. Fig 1 shows a flow diagram indicating the reasons for exclusion from the trial. The results of the primary analysis of the study have been published.[17] Secondary endpoints of the study were to assess relationships with long-term outcomes for: baseline F_p_ of the primary lesion and / or largest regional lymph node; the change in F_p_ of the primary lesion and / or largest regional lymph node between the baseline DCE-MRI scan and the second DCE-MRI scan (post two cycles of ICT); and RECIST response between the baseline and second DCE-MRI scan (post two cycles of ICT) using target lesions from both the primary tumour and nodal metastases as per RECIST guidelines version 1.1. [25]

### Statistical analysis

IBM SPSS Statistics 22 (IBM, Armonk, USA) was used for all statistical analyses. Patients were stratified by median F_p_ of the primary tumour and by the largest regional lymph node. Kaplan-Meier curves were plotted for these groups with respect to overall survival (OS), disease-specific survival (DSS) and locoregional control (LRC) measured from date of first day of treatment. F_p_ values were also determined in patients undergoing the second DCE-MRI scan (after two cycles of ICT). Pre-treatment values were then subtracted to obtain a value for the change in F_p_. Patients were again stratified by median F_p_ of the primary tumour and largest regional lymph node. Patients were also stratified by RECIST response defined as complete or partial (CR or PR) versus stable or progressive disease (SD or PD). Cox regression was used to assess differences in outcome between all groups.

## Results

Table 1 summarizes the patient characteristics for the group. Median follow-up in surviving patients was 36 months (range 23–49 months). ICT was stopped and treatment changed to palliation in one patient following identification of pre-treatment lung metastases. This patient was included in the OS analyses (on an intention to treat basis) but was excluded from the DSS and LRC analyses.

**Table 1:** Trial patient and tumour characteristics.

Characteristic		Number	Percent (%)
**Age (years)**	Median (range)	56 (38–73)	-
**Gender**	Male	38	90.5
	Female	4	9.5
**Primary site**	Oral cavity	2	4.8
	Oropharynx	31	73.8
	Nasopharynx	2	4.8
	Hypopharynx	7	16.7
**Grade**	Moderately differentiated	20	47.6
	Poorly differentiated	17	40.5
	Unknown	5	11.9
**Tumour classification^a^**	T2	13	31.0
	T3	14	33.3
	T4	15	35.7
**Node classification^a^**	N0	3	7.1
	N1	1	2.4
	N2	34	81.0
	(N2b)	(22)	52.4
	(N2c)	(12)	28.6
	N3	4	95.2
**Stage^a^**	Iva	37	88.1
	IVb	5	11.9
**IMRT**	Yes	41	97.6
	No	1	2.4
**WHO performance status**	0	31	73.8
	1	10	23.8
	2	1	2.4
**P16 status (whole group)**	Positive	30	71.4
	Negative	10	23.8
	Unknown	2	4.8
**P16 status (oropharynx)**	Positive	27	87.1
	Negative	2	12.9
	Unknown	2	12.9
**Smoking Status**	Never	9	21.4
	Ex </ = 1 year	8	19.0
	Ex > 1 year	15	35.7
	Current	10	23.8

IMRT: Intensity-modulated radiation therapy; WHO: World Health Organisation; HPV: Human papilloma virus; ISH: In situ hybridization.

^a^According to American Joint Committee on Cancer staging [21].

**Fig 2:**
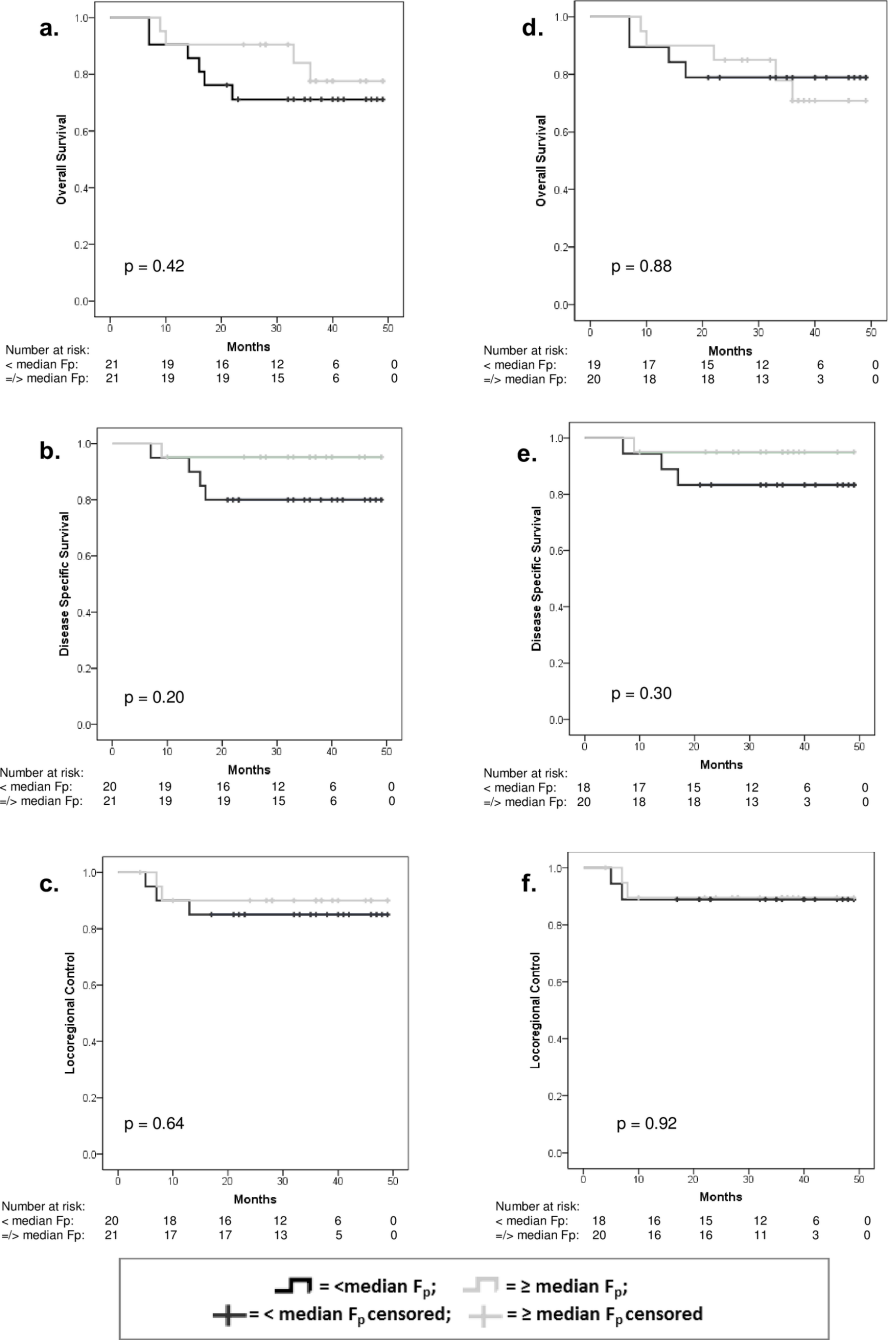
Tumour plasma perfusion (F_p_) in relation to overall survival (a), disease specific survival (b) and locoregional control (c). Nodal plasma perfusion in relation to survival (d) disease specific survival (e) and locoregional control (f).

Median survival was not calculated because a 50% event rate was not reached for any group. At a median 3-year follow up OS for the whole group was 74.1%, DSS 87.6% and LRC 87.4%. Median tumour F_p_ was 43.8 ml/100ml/min, interquartile range 25.5–81.5 ml/100ml/min. As shown in Table 2, there were no statistically significant differences in OS (p = 0.42), DSS (p = 0.20) or LRC (p = 0.64) for patients with low versus high pre-treatment tumour F_p_. Median nodal F_p_ was 28.19ml/100ml/min, interquartile range 18.01–55.20 ml/100ml/min. There were no statistically significant differences in OS (p = 0.88), DSS (p = 0.30) or LRC (p = 0.92) for patients with low versus high nodal Fp. Fig 2 shows the Kaplan-Meier curves for tumour and nodal F_p_ versus OS, DSS and LRC.

**Table 2:** Results from univariable Cox regression analyses.

Outcome measure (frequency^a^)	Overall survival	P value	Disease specific	P value	Locoregional control	P value
	HR (95% CI)		survival HR (95% CI)		HR (95% CI)	
**Tumour**						
**<median (21)**	1		1		1	
**≥median (21)**	0.59 (0.17–2.10)	0.42	0.24 (0.03–2.10)	0.20	0.65 (0.11–3.91)	0.64
**Nodal Fp**						
**<median (19)**	1		1		1	
**≥median (20)**	1.11 (0.30–4.13)	0.88	0.30 (0.03–2.87)	0.30	0.91 (0.13–6.43)	0.92
**Change in**						
**tumour Fp**						
**<median (18)**	1		1		1	
**≥median (19)**	1.05 (0.28–3.91)	0.95	0.01 (0.00–44.91)	0.30	0.25 (0.03–2.21	0.21
**Change in**						
**nodal Fp**						
**<median (16)**	1		1		1	
**≥median (16**	0.55(0.13–2.30)	0.41	0.49 (0.04–5.38)	0.56	0.95 (0.13–6.73)	0.95
**RECIST response**						
**CR or PR (24)**	1		1		1	
**SD (13)**	0.95 (0.24–3.80)	0.94	0.61 (0.06–5.86)	0.66	0.44 (0.05–3.90)	0.44

^a^One patient included in the overall survival (OS) group on an intention to treat basis was not included in the DSS and LRC analyses. This patient was excluded from further analyses as it became apparent during induction chemotherapy that the patient had distant metastases. Induction chemotherapy was abandoned and treatment was swapped to palliative.

Change in primary tumour F_p_ was available for 37 patients and change in nodal F_p_ for 32 patients. Using Cox regression analysis, changes in F_p_ between the pre-treatment DCE-MRI and the DCE-MRI post two cycles of ICT had no prognostic significance (Table 2).

**Fig 3:**
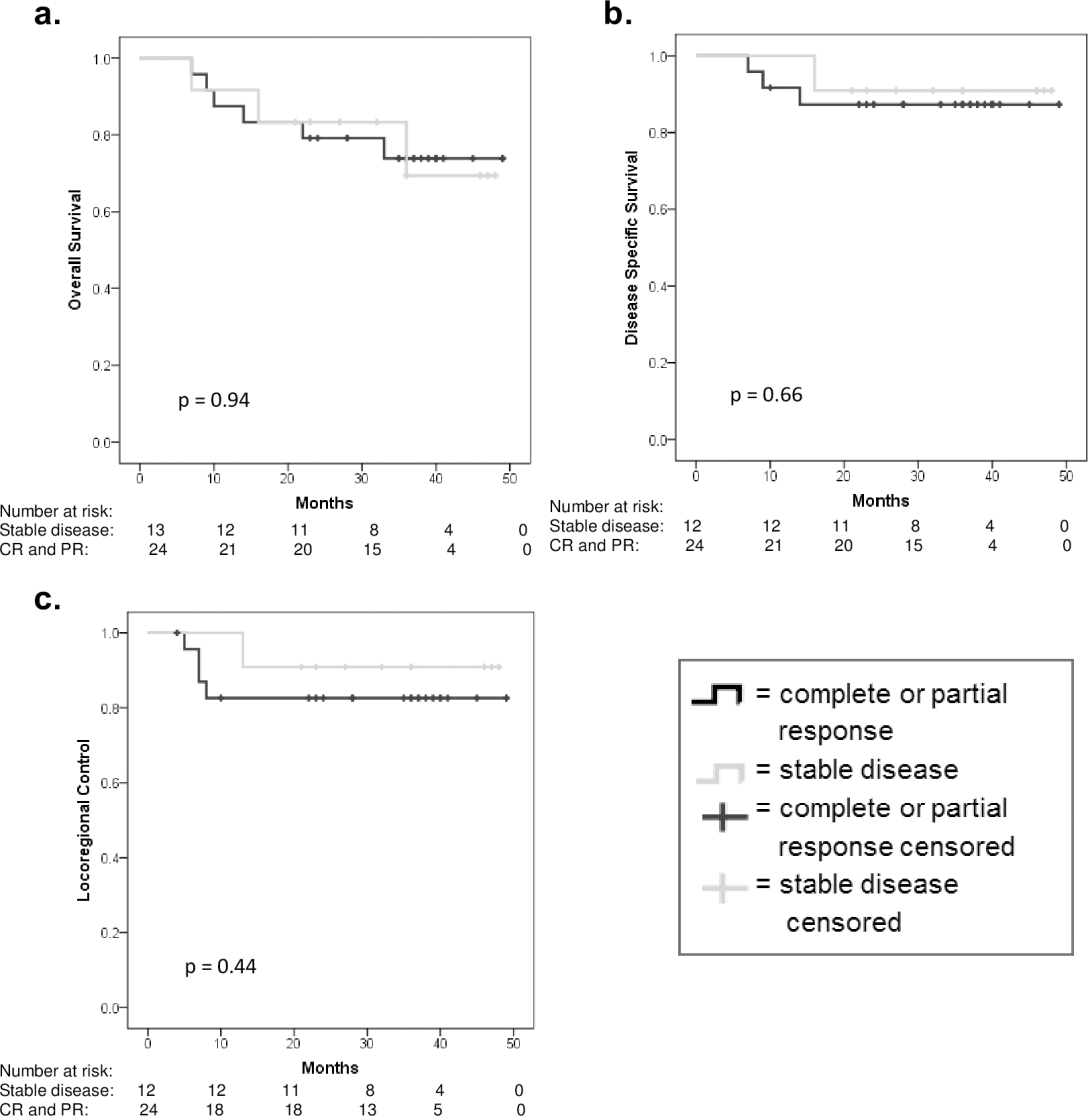
RECIST response to induction chemotherapy in relation to overall survival (a), disease specific survival (b) and locoregional control (c).

RECIST response between the pre-treatment MRI and the MRI post two cycles of ICT was available in 37 patients. One patient had CR, 23 patients had PR, 13 patients had SD and no patients had PD. The outcomes for patients with a CR or PR versus SD were not statistically significant for any outcome measures as shown in Table 2. Fig 3 shows Kaplan-Meier curves for RECIST response versus outcome.

Results from univariable analysis of smoking status, performance status,[26] p16 status, tumour differentiation and nodal grade with regards to survival outcome are beyond the scope of this article but can be found in supporting information S1 Table.

## Discussion

The primary analysis of the study showed that high pre-treatment tumour F_p_ evaluated using pre-treatment DCE-MRI imaging in high stage HNSCC predicts initial response to ICT.[17] The secondary analysis reported here showed that this early prediction of good response to ICT did not correlate with good long term outcomes. Also, neither change in plasma perfusion post two cycles of ICT nor RECIST response were prognostic for long-term survival outcomes.

The tumour microenvironment affects the delivery and penetration of chemotherapy to malignant cells and so impaired tumour perfusion leads to a poor treatment response.[27–29] It is therefore reasonable to expect that F_p_ (F_p_ is equivalent to blood flow [BF] adjusted for haematocrit) might be prognostic for long term outcomes. Indeed, several HNSCC studies show pre-treatment tumour hypoxia and haemodynamic imaging parameters such as blood volume (BV) and BF are prognostic for survival outcomes [30–37] although there are conflicting reports.[38] However, these studies were performed on patients receiving definitive locoregional treatment such as XRT or CRT rather than ICT. Published trials assessing perfusion parameters relating to ICT predominantly report initial response to ICT treatment only.[18–20] Hence, whilst there is evidence that tumour perfusion parameters can predict immediate response to ICT, ours is one of the first studies to assess long term outcomes. Despite pre-treatment Fp predicting initial response to ICT,[17] it does not equate to long term survival benefit. This result was echoed by Bisdas et al who found that baseline BV and BF predicted initial ICT response. However, it was noted that progression free survival in the responder group was not significantly different to the non-responders group (p = 0.80).[39] Nevertheless, stratifying patients to different treatments based on initial ICT response is currently performed in clinical practice. The RTOG 91–11 study [40] designed a trial modelled on the Department of Veterans Affairs Laryngeal Cancer Study Group trial of 1991 where patients in the ICT arm received either a further cycle of ICT then radiotherapy or salvage surgery depending on their response post 2 cycles of ICT. Urba et al 2006 [41] also suggested that single cycle ICT selects patients for organ sparing treatment rather than total laryngectomy depending on initial ICT response. There are differences in what constitutes a good or poor response to treatment ranging from “no response” to “50% response” to a “major response” and varying numbers of preliminary cycles of ICT given. In clinical practice, initial response to ICT is thought to suggest a tumour’s inherent treatment responsiveness and is considered an aid for subsequent treatment decisions. Our study suggests, however, that this practice of using response to ICT as a tool to stratify patients to different locoregional treatments may need to be revisited as an inadequate response to ICT may not translate into a poor outcome post CRT.[41] Larger trials are needed to verify our results.

A possible explanation for the lack of prognostic significance might be intra-tumoural heterogeneity. Tumours can have sub-regions with variable blood flow, architecture, metabolism, cell proliferation, genotypes and phenotypes.[42–45] Gerlinger et al reported that “gene expression signatures of poor and good prognosis were detected in different regions of the same tumour”.[44] ICT is used predominantly to tackle distant micrometastases as an adjunct to definitive treatment and although potentially producing considerable tumour and nodal shrinkage, there is only a complete response in 0–40% of patients.[17, 20] It may be that the prognostic features of the remaining tissue are what ultimately determine the response to locoregional treatment and survival outcome. This may explain some of the discrepancies between findings based on patients treated solely with locoregional treatment of (chemo)radiotherapy and those treated with ICT first which best tackles distant metastases.

In 1996 the Food and Drug Administration stated that more cancer drugs would be granted accelerated approval if their benefit could be demonstrated by objective evidence of tumour shrinkage.[46] It was noted that evidence of better survival could now be demonstrated later implying that tumour shrinkage and survival benefit were related. There is evidence that in several cancer sites that this is the case.[47, 48] Studies including a meta-analysis by El-Maraghi and Eisenhauer showed that objective tumour response was a useful endpoint in phase II trials for several solid tumours as its observation predicted for eventual success in phase III trials.[49, 50] However, HNSCC were not included in these trials and the result was not seen in all subsites studied.

The relationship between objective tumour shrinkage (RECIST response) and long term outcomes has not been extensively studied in HNSCC but, like our study, those performed in HNSCC suggest RECIST response cannot be used a surrogate marker for long term survival outcomes. Passero et al found no relationship between a complete RECIST response measured by CT and progression free survival (PFS) in 53 HNSCC patients treated with concurrent CRT +/- ICT.[51] Matoba et al found no relationship between RECIST response measured using MR prior to and eight weeks post CRT on locoregional control or survival.[52] Studies have shown that RECIST response measured by CT does not correlate with pathological response in HNSCC, which may explain why RECIST response is an unreliable predictor of long-term outcome.[53, 54] Hence, although a widely used method of measuring change in tumour size in regular clinical use and as part of the reporting of trial outcomes, RECIST response appears to have no value as a prognostic factor for long-term survival outcomes in HNSCC. This finding has practical implications as RECIST response is used frequently in clinical practice and trial reporting despite a lack of evidence supporting its use for HNSCC. There is, therefore, a need for larger clinical trials investigating initial RECIST response to treatment in relation to long term survival outcomes, which need to investigate the effect of the treatment regimen used, i.e. CRT alone or with additional ICT.

Limitations of this study are that it is single-centre and non-randomized. Also, the study was powered to assess the relationship between pre-treatment DCE-MRI parameters and RECIST response rather than relationships with long-term outcomes. Strengths are that it is prospective and focuses on the patient group most likely to benefit from TPF ICT, i.e., those with stage IV disease (and hence high risk of distant metastases). This cohort, therefore, is a representative group of patients that is most likely to be offered this treatment regimen.

Regarding future studies, larger trials are required to validate our study results in relation to using initial ICT response and RECIST response as an aid to treatment stratification. Further studies into intra-tumour heterogeneity and how it correlates with outcomes following ICT are also required.

## Conclusions

Pre-treatment tumour F_p_, change in tumour F_p_ measured using DCE-MRI and RECIST response post two cycles of ICT are not prognostic for long-term survival outcome in HNSCC patients treated with ICT followed by chemotherapy and IMRT. Intra-tumour heterogeneity post ICT may explain the inability to predict long-term outcomes prior to treatment. RECIST response to ICT may not be an appropriate endpoint to determine efficacy of treatment in a clinical or phase II trial setting for HNSCC patients.

## Supporting information

S1 Table Results from univariable analyses using Cox regression. (DOCX)
