# Supplementary material for: Pre-treatment tumour perfusion parameters and initial RECIST response do not predict long-term survival outcomes for patients with head and neck squamous cell carcinoma treated with induction chemotherapy
Source: PLoS One. 2018 Mar 28;13(3):e0194841. doi: 10.1371/journal.pone.0194841 (PMC5874054; doi:10.1371/journal.pone.0194841)
Supplement: S1 Table — (DOCX) [file pone.0194841.s001.docx]

# Supporting information

**S1 Table: Results from univariable analyses using Cox regression.**

| Univariate analysis  (frequency^a^) | Overall survival  HR (95% CI) | P value | | Disease specific survival  HR (95% CI) | P value | Locoregional control  HR (95% CI) | P value |
| --- | --- | --- | --- | --- | --- | --- | --- |
| Smoking status  Never or ex > 1 year (24^a^)  Current or ex ≤ 1 year (18) | 1  1.56 (0.45-5.38) | | 0.49 | 1  5.63 (0.63-50.41) | 0.08 | 1  5.89 (0.66-52.75) | 0.07 |
| P16 status  Negative (10)  Positive (30) | 1  0.20 (0.05-0.75) | | ***0.01*** | 1  0.08 (0.01-0.81) | **0.01** | 1  0.00 (0.00-4455.98) | ***<0.0005*** |
| Tumour differentiation  Moderate (20)  Poor (17) | 1  0.13 (0.02-1.00) | | ***0.02*** | 1  0.02 (0.00-22.11) | ***0.03*** | 1  0.02 (0.00-21.52) | ***0.03*** |
| WHO performance status^b^  0 (31)  1-2 (11) | 1  6.16 (1.72-22.04) | | ***0.00*** | 1  5.78 (0.96-34.72) | ***0.03*** | 1  2.29 (0.38-13.75) | 0.35 |
| Nodal status^c^  N0, N1, N2a, N2b (26)  N2c, N3 (16) | 1  1.12 (0.31-3.96) | | 0.87 | 1  0.43 (0.47-3.80) | 0.43 | 1  1.14 (0.19-6.84) | 0.88 |

WHO: World Health Organisation. Statistically significant p values are highlighted in bold italic. ^a^One patient included in the overall survival (OS) group on an intention to treat basis was not included in the DSS and LRC analyses. This patient was excluded from further analyses as it became apparent during induction chemotherapy that the patient had distant metastases. Induction chemotherapy was abandoned and treatment was swapped to palliative. ^b^(1). ^c^According to American Joint Committee on Cancer staging.(2)

1. Oken MM, Creech RH, Tormey DC, Horton J, Davis TE, McFadden ET, et al. Toxicity and response criteria of the Eastern Cooperative Oncology Group. American journal of clinical oncology. 1982;5(6):649-55.

2. Edge SE, Byrd DR, Compton CC, Fritz AG, Greene FL, Trotti A. AJCC Cancer Staging Manual. 7th edition ed. New York, USA: Spinger; 2009.
